# Editorial: Internet pharmacies and the online pharmacy market: trends, perspectives and challenges

**DOI:** 10.3389/fphar.2024.1489396

**Published:** 2024-09-25

**Authors:** András Fittler, Norah Othman Abanmy, Anna Serefko, Inayat Ur Rehman, Róbert György Vida

**Affiliations:** ^1^ Department of Pharmaceutics, Faculty of Pharmacy, University of Pécs, Pécs, Hungary; ^2^ Department of Clinical Pharmacy, College of Pharmacy, King Saud University, Riyadh, Saudi Arabia; ^3^ Department of Clinical Pharmacy and Pharmaceutical Care, Medical University of Lublin, Lublin, Poland; ^4^ Department of Clinical Pharmacy and Pharmacy Practice, Universiti Malaya, Kuala Lumpur, Malaysia

**Keywords:** internet pharmacies, medication safety, supply chain and risk, online medicinal products, consumer attitude and behaviour, quality assurance, regulation and guidance

The online pharmacy market has emerged as a rapidly growing channel in the pharmaceutical supply chain since the turn of the century (Long et al., 2022; Fittler et al., 2023). Despite its growth, the true size of the market remains elusive, with thousands of internet pharmacies accessible worldwide (Orizio et al., 2011). While purchasing medication from legitimate online pharmacies is an accepted practice in developed countries, many studies around the world have documented significant changes in consumer demand for distance buying of medicines (Lobuteva et al., 2022). The market is plagued by illegitimate vendors (also referred to as illicit or rogue online pharmacies), posing significant risks to patient safety (Limbu and Huhmann, 2023; Mackey and Nayyar, 2016; Ashraf et al., 2024; Ozawa et al., 2022). This Research Topic aimed to frame the trends, perspectives and methodological considerations of the challenges associated with internet pharmacies and the online pharmacy market in the submitted articles. The key themes that emerged were market trends and consumer attitudes; the current and ongoing challenges related to quality assurance and regulation; and the urgent need for a multi-stakeholder collaborative approach to resolve the issues related to this complex pharmaceutical supply channel.

Trends in the use of online pharmacies and consumer attitudes have changed; consequently, online purchases of medication and health products are becoming more prevalent in numerous regions. The COVID-19 pandemic has significantly accelerated the shift to online pharmacies, and a study by Fittler et al. focusing on Central European countries highlighted a significant increase in the frequency of online medicine purchases. However, despite this growth, the traditional “offline” drug supply chain will not be replaced by online platforms as most consumers still prefer traditional pharmacies, indicating continued trust in brick-and-mortar establishments. A systematic review by Almomani et al. explored the reasons why consumers opt to purchase prescription medicines online and identified factors such as convenience, cost, and privacy as the main reasons. Twelve drivers were detected by Limbu et al. in a review that shed light on the factors that encourage customers to buy medication online. Higher-income consumers, White American consumers, and more educated consumers were the most liable to such purchases. The existing literature covered in this review included 47 papers from 29 countries in Europe, North America, Australia, Asia, the Arabian Peninsula, and Africa, although published studies carried out in South America are lacking. Despite the readily observable benefits, the risks associated with purchasing medicines over the internet must also be considered, including the potential for counterfeit medicines and the lack of professional healthcare oversight. Accordingly, increased consumer awareness and regulatory measures are necessary to ensure safe online purchasing practices.

The first online pharmacies were established in 1999 (Mäkinen et al., 2005) but regulatory and quality assurance challenges are persistent. Consequently, numerous problems related to online health product purchases remain unresolved for a quarter of a decade. The challenges of regulating online pharmacies are compounded by the global nature of the internet, varying national laws, and the complexity of the product categories offered for sale. A UK-based study by Oriakhi et al. investigated the operation of online pharmacies, especially during the COVID-19 pandemic, revealing significant patient safety risks due to the prevalence of “rogue” pharmacies. This study found that 47% of the 116 online pharmacies assessed were rogue, often selling prescription-only medicines (POMs) without a prescription or consultation and offering controlled medicines like alprazolam and diazepam. A study by Vida et al. on the safety and risks associated with cannabidiol (CBD) oils purchased online revealed quality Research Topic related to discrepancies in CBD content and misleading health claims, pointing to broader Research Topic related to patient safety. In addition to medications and dietary supplements, a study conducted by Garcia et al. on the online availability of anabolic agents in sports (e.g., oxandrolone, DHEA, and androstenedione) revealed that these substances are also easily accessible online, often without a prescription. Chereches et al. evaluated the role of online pharmacies in providing Food for Special Medical Purposes (FSMPs) based on a survey of cancer patients in Romania. The study revealed patient satisfaction with access, concerns about cost, and the need for information provided by healthcare professionals during FSMP selection. The authors underlined that digital solutions should complement and enhance, rather than replace, the traditional interactions and expert advice provided by healthcare providers. One of the most important aspects of successful online pharmacy services is the application of a strong regulatory environment to regulate and prevent fraudulent transactions. Although the United States, the UK, Canada, Europe, and Australia have developed and implemented clear and comprehensive regulations, such measures are still being established in other parts of the world, such as the Gulf region. Alfageh et al. reviewed the regulations in Saudi Arabia, Kuwait, the United Arab Emirates, Qatar, Bahrain, and Oman and found that the legal framework for internet pharmacies is somewhat comparable among the surveyed countries; however, it is still in its infancy, and online pharmacy service providers have limited awareness of the existing regulations. The authors of this paper emphasize the importance of establishing firm regulations by local authorities in the Gulf region that control online pharmacy services locally, in addition to the importation of medication for personal use. These findings highlight the urgent need for international harmonization of regulations and the establishment of robust and transnational verification systems to protect consumers.

A multi-stakeholder approach and complex risk assessment methodologies are required to address the challenges of the online pharmacy market and guarantee its safety (Vida et al., 2020). This involves collaboration among organizations, patients, manufacturers, distributors, authorities, and search engine providers to ensure the integrity of the supply chain (Fittler et al., 2022). By working together, these stakeholders can maximize the benefits of online pharmacies while minimizing the associated risks.

In conclusion, the online pharmacy market has emerged as a dynamic and rapidly evolving segment of the pharmaceutical supply chain that is significantly transforming how consumers access medications. This supply channel and the products offered to patients are unique in numerous aspects, including safety, efficacy, quality, regulation, and vulnerability. The contributions to this Research Topic provide valuable insights into the dynamics of the online pharmacy market and provide further evidence to create a take-home message that should be clear to all stakeholders, including regulators, health professionals, authorities, search engines and internet providers, and patients: “We must find the balance between convenience and safety”. As the market continues to evolve, supporting research that facilitates the publication of studies from different countries and disciplines is imperative, ultimately contributing to safer and more effective online pharmaceutical practices. Future research should focus on opportunities for developing global verification systems, enhancing quality assurance, establishing effective and ethical marketing practices, implementing and evaluating public awareness and education campaigns, developing and evaluating online pharmacy/telepharmacy services, and strengthening law enforcement to protect consumers in this rapidly evolving market and ultimately advance digital health services.
